# Molecular models for intrastrand DNA G-quadruplexes

**DOI:** 10.1186/1472-6807-9-64

**Published:** 2009-10-07

**Authors:** Federico Fogolari, Haritha Haridas, Alessandra Corazza, Paolo Viglino, Davide Corà, Michele Caselle, Gennaro Esposito, Luigi E Xodo

**Affiliations:** 1 Dipartimento di Scienze e Tecnologie Biomediche, Università di Udine, Piazzale Kolbe 4 - 33100 Udine, Italy; 2 Istituto Nazionale Biostrutture e Biosistemi, Viale Medaglie d'Oro 305, 00136 Roma, Italy; 3 Dipartimento di Fisica Teorica Università di Torino, Via P. Giuria 1 10125 Torino, Italy

## Abstract

**Background:**

Independent surveys of human gene promoter regions have demonstrated an overrepresentation of G_3_X_*n*1_G3X_*n*2_G_3_X_*n*3_G_3 _motifs which are known to be capable of forming intrastrand quadruple helix structures. In spite of the widely recognized importance of G-quadruplex structures in gene regulation and growing interest around this unusual DNA structure, there are at present only few such structures available in the Nucleic Acid Database. In the present work we generate by molecular modeling feasible G-quadruplex structures which may be useful for interpretation of experimental data.

**Results:**

We have used all quadruplex DNA structures deposited in the Nucleic Acid Database in order to select a list of fragments entailing a strand of three adjacent G's paired with another strand of three adjacent G's separated by a loop of one to four residues. These fragments were further clustered and representative fragments were finally selected. Further fragments were generated by assemblying the two strands of each fragment with loops from different fragments whenever the anchor G's were superimposable. The fragments were used to assemble G quadruplex based on a superimposability criterion.

**Conclusion:**

Molecular models have been generated for a large number of G_3_X_*n*1_G_3_X_*n*2_G3X_*n*3_G_3 _sequences. For a given sequence not all topologies are possible with the available repertoire of fragments due to steric hindrance and low superimposability. Since all molecular models are generated by fragments coming from observed quadruplex structures, molecular models are in principle reliable and may be used for interpretation of experimental data. Some examples of applications are given.

## Background

It is generally recognized that in addition to the canonical Watson-Crick double-stranded conformation, DNA can assume a variety of secondary structures including triplex [1-3], cruciform [4], quadruplex [5-7] and Z-DNA [8]. Quadruplex DNA, also called G4-DNA, is stabilized by G-quartets, planar arrays of four guanines paired by Hoogsteen hydrogen bonding, and monovalent alkali cation, K+ or Na+, located in the central cavity of the structure. G-quartets can stabilize a variety of quadruplex structures which can be intermolecular or intramolecular, in which single-stranded DNA is folded to provide the four strands of the guanine scaffold. In the human genome the sites that can potentially form G4-DNA are estimated to be more than 300.000. They are not randomly distributed, but located preferentially in repetitive genomic sequences such as the telomeres, ribosomal DNA and the immunoglobulin heavy-chain switch regions [7]. Moreover, G-rich sequences have been found with a high frequency in the control regions of proto-oncogenes, either upstream or downstream the transcription start site (TSS) [9]. While the formation of G4-DNA structures in the 5' overhang of the telomeres has the function of reducing the effect of endogenous nucleases and stabilizing the chromosomes, the possible role of G4-DNA in the promoter of proto-oncogenes is still a matter of debate. The observation that some common transcription factors including SP1 (binding site: RGGCGKR), KLF (binding site: GGGGTGGGG), and MAZ (binding site: GGGAGGG), recognize regions composed by runs of guanines, potentially capable to extrude G4-DNA, raises the hypothesis that this unusual structure may be somehow involved in transcription regulation. Hurley and co-workers reported that a G-rich element (-142 to -115 bp) upstream of the major P1 promoter folds into a stable G-quadruplex [10]. As G > A point mutations abrogating the capacity of the promoter to form a quadruplex enhance transcription, while porphyrinic ligands that stabilize G4-DNA reduce transcription, it was concluded that quadruplex DNA should behave as a repressor. Such mechanism has been hypothesized also for other proto-oncogenes including KRAS [11-13], CKIT [14], VEGF [15], CMYB [16], Rb [17] and BCL-2 [18,19]. Nucleic acids structures are difficult to probe in vivo, and the main evidence that G4-DNA exists in cells is that antibodies raised against G-quadruplex DNA label the macronuclei of a ciliate [20]. Furthermore, the observation that several prokaryotic and eukaryotic proteins recognize and bind to quadruplex DNA [21] also supports indirectly that it exists in vivo. Some of these proteins, hnRNP A1 [22], POT-1 [23] and human Werner syndrome helicase [24] have also resolvase activity against this structure.

Given its biological importance, G-quadruplex structures have become target for several drug design studies (see e.g. [6,25-28]). Many efforts have been made to resolve by crystallography or NMR the structure of quadruplex DNA. However, so far a limited number of structures has been resolved, mainly because G-rich sequences at high concentrations tend to assume a variety of inter-molecular and intra-molecular structures. So, molecular modeling can be very helpful to get insight into putative G4-DNA structures formed by biological relevant sites.

In particular, there is a widespread interest in sequences possessing the motif G_3+_X_*n*1_G_3+_X_*n*2_G_3+_X_*n*3_G_3+_, where G_3+ _indicates 3 or more G's and *n*1, *n*2 and *n*3 are numbers greater than one. These sequences have been demonstrated to be able to form intrastrand G-quadruplexes [5,25,29-39].

Structure determination of intrastrand G-quadruplex has been elusive, because of the observed conformational equilibria which are detrimental for both NMR and X-ray crystallographic studies. Indeed, base modifications have been used to stabilize a particular conformation and more in general it has been reported that only one out of several tens of starting G-quadruplex putative sequences are amenable to structural study [5]. To the best of our knowledge there are only thirteen intrastrand G-quadruplex structures solved which do not contain modified bases.

When this figure is compared with the number of potential G-quadruplexes identified around the TSS of genes and involved in gene regulation by independent studies [40-48] the enormous gap between sequence and structure studies is apparent.

Besides the possibility that the same sequence could adopt more conformations, which could prevent structure resolution, the high concentration typically required for structural methods could favor intermolecular assembly over intramolecular formation of G-quadruplexes. Intermolecular G-quadruplexes (dimers or tetramers) are roughly ten times more represented in the Nucleic Acid Database (NDB) [49] or Protein Data Bank (PDB) [50] than intramolecular G-quadruplexes.

However, the 3D structure of nucleic acids can be inferred from sequence and indeed a pipeline of RNA secondary structure prediction and structure reconstruction has been recently shown to predict RNA structures with high accuracy [51-53]. The quality of the putative models relies on the quality of RNA secondary structure prediction.

For G-quadruplexes the complexity of possible topologies and the limited repertoire of structures solved makes this task much more difficult. The MC-Fold and MC-sym prediction pipeline proceeds from a single sequence to a single structural model determined according to restraints derived from structural prediction [51].

In this work we proceed in a different way, i.e. we simply explore what conformations could be assembled by the repertoire of observed fragments in a dataset of quadruplex structures. The rationale behind this study is that the latter dataset entails the most stable structural features of G-quadruplexes. It is reasonable to expect that a predictive model incorporating features found in this dataset should be stable. We assemble novel quadruplex structures by assembling combinatorially all fragments encoding for strands participating in the G-quadruplex stems and loops connecting two strands of the G-quadruplex. The set of predictive models is instructive in that it highlights those topologies and loop lengths which can be combined to assemble a model together with their frequencies.

The method is inspired by the program MC-Sym [51-53] which, combined with the secondary stucture prediction program MC-Fold was able to accurately predict RNA structure starting from a dataset of fragments. The program assembles the fragments in a hierarchical manner, subject to constraints and retaining all or only the best fragments generated at each step [51]. The program has many options to control the number of fragments kept at each step of the building procedure and is designed to achieve accuracy and efficiency.

No energy or scoring function is used on the contrary here because the constraints imposed by the quadruplex structure are sufficient to efficiently counterbalance the number of conformations assembled combinatorially from the starting fragments.

We determine a library of 4418 structures (and sequences), further refined by energy minimization, which cover more than half of the possible topologies. The structures are grouped together according to unique glycosidic bond conformation, topology and loop length and for each group the most representative structure is chosen. This clustering procedure results in a set of 116 representative G-quadruplex structures which can be used, in the absence of other structural information, to interpret data like those coming from UV, CD or FRET experiments which provide only partial structural information. Examples of possible applications are given.

## Results and Discussion

### G-quadruplex model generation

#### Quadruplex structure selection

The search in the Nucleic Acid Database (NDB) [49] for quadruplex DNA structures returned 101 entries. Unfortunately this list did not include all G-quadruplex containing structures. The Protein Data Bank (PDB) [50] was searched for entries containing the words "tetraplex" or "quadruplex" and the list was filtered by visual inspection. The sequences extracted for each chain in the corresponding PDB files were searched for a G_3_X_*n*1_G_3_X_*n*2_G_3_X_*n*3_G_3 _motif. Only 14 such sequences were found that were corresponding to intrastrand G-quadruplexes (PDB ids. 143D, 186D, 1KF1, 1XAV, 230D, 2F8U, 2GKU, 2HY9, 2JPZ, 2JSL, 2JSM, 2O3M, 201D, 3CDM), including 230D which contains the nucleotides uridine and inosine-phosphate. A literature survey was also done independently to retrieve the released intramolecular quadruplex structures. The search query ((quadruplex OR tetrad OR tetraplex OR G-4 OR tetramer) AND (intramolecular OR unimolecular OR monomolecular)) in Pubmed resulted in 344 hits. Scanning the abstracts manually resulted in 86 articles relevant to structural studies of quadruplexes. The author names from these articles were collected and searched for individually in Nucleic Acid Database for any deposited quadruple helix structures. No novel intrastrand structure was found in this procedure and thus we trust the set of 14 structures to be complete.

Such paucity of intrastrand G-quadruplex structure may be related to the well known polymorphism of poly-dG [54,55] and the difficulty in obtaining crystals for longer DNA sequences or obtaining single solution forms for NMR studies [5].

#### Assembly of DNA G-quadruplex stems from fragments

The selection of fragments from the available structures produced, after clustering and selection of representatives for similar conformations, 58 stem fragments and 65 loop fragments, each representing different features, with respect to diversity in sequence, parallel or antiparallel arrangement, loop length and base pairings.

We use here the term "base pairing" as possible participation in the same G-tetrad. The base pairings of the first base in the fragment may involve the edge of the base involved in Watson-Crick base pairing (entailing atoms N1 and N2) or the edge of the base which is involved in Hoogsteen base pairing (entailing atom O6 and N7). These base pairs are hereafter named edge-wise. Alternatively hydrogen bonds may be missing altogether when the second stem is located at the opposite corner of the tetrad. These base pairs will be hereafter named tip-wise. We refer to the three possibilities mentioned above as WH, HW or X (cross) pairing, respectively, or for the sake of notation 0, 2 and 1, respectively.

Loops connecting edge-wise and tip-wise antiparallel strands correspond to lateral and diagonal loops, respectively according to Webba da Silva [56]. Edge-wise loops connecting parallel strands correspond to propeller loops, according to the same author.

The distribution in loop lengths is uneven, with just two loops of length 2 and seven loops of length 1. These short loops are found exclusively in a parallel arrangement. 3 and 4 nucleotide loops are found 35 and 22 times, respectively. Longer loops are found both parallel and antiparallel. Interestingly, a loop connecting two parallel strands at the opposite corners of a tetrad is also present. Bases in this loop, however, participate the G-tetrads and therefore will be discarded, for steric reasons, in the following assembly of G-quadruplexes.

The features of the selected fragments are reported in Table 1.

**Table 1:** Non-redundant features of the fragments selected from the database.

**syn/anti**	**a/p**	**loop length**	**pairing**	**counts**
a s a	a	4	111	13

a a a	p	3	000	10

s a a	a	3	200	7

s s a	a	3	002	6

s a a	p	3	200	6

a a a	p	1	000	6

a s a	a	4	020	5

s s a	a	3	220	2

a s a	a	3	020	2

a a a	p	4	111	2

s s a	a	4	111	1

s a s	a	3	111	1

s a a	p	2	200	1

s a a	p	1	200	1

s a a	a	4	200	1

s a a	a	4	111	1

a s a	a	4	202	1

a s a	a	3	202	1

a a a	p	2	000	1

syn/anti indicates the conformation at the glycosidic bond of the first three G's, a/p indicates antiparallel/parallel arrangement, 0, 1 and 2 pairings are described in the text and the number of fragments different in sequence or conformation is given in the column "counts".

The stems of the fragments were used to build up the four-strand G-quadruplex stems. With the loose requirements of no more than 0.8 Å RMSD between the superimposing fragments and no overlap below 0.5 times the sum of van der Waals radii (see Methods) 646 G-quadruplexes were built whose tetrad planes were rebuilt using the frame provided by the first three G strand in the sequence. In this step it was checked that the model G-tetrad could be well placed on the C1' anchor points. Models which exhibited an RMSD larger than 3.0 Å were discarded, leaving a set of 509 G-quadruplex stem models.

**Figure 1:**
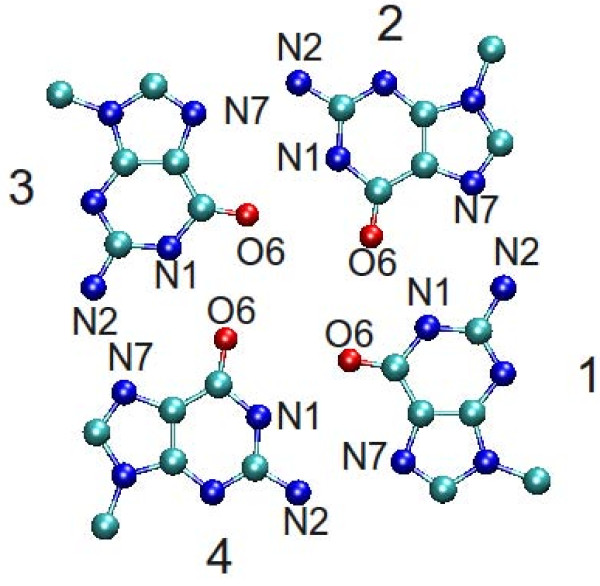
**The standard G-tetrad with reference numbering**. When the glycosidic bond angle is *anti *the chain progresses over the page, when it is syn the chain progresses below the page. According to Webba da Silva [56] the sign of the loop topology is positive when the first stem is progressing towards the viewer and the second stem is found rotating clockwise, and negative when it is found rotating anti-clockwise. E. g., when the glycosidic bond angle is *anti *the topology of a loop connecting the stem of base 1 to the stem of base 2 would be marked with - sign, and the topology of a loop connecting the stem of base 1 to the stem of base 4 would be marked with + sign.

Rebuilding the G-tetrad was necessary because, due to the tolerant cutoff used for fragment assembly, base pairing was not always consistent with the hydrogen bonding pattern of a G-tetrad. For this reason the four G's constituting the G-tetrad were replaced by a standard G-tetrad by first superimposing the first G (numbered 1 in Figure 1) on the G of the first strand in the molecule in order to determine the orientation of the G-tetrad and then superimposing the C1' atoms of the tetrad with those of the G-quadruplex.

#### Assembly of DNA G-quadruplex from G-quadruplex stems and loops

The strands of the 509 G-quadruplex stems determined as described above were connected using the loops of the fragments selected from the NDB and PDB quadruplex dataset. 65 non-redundant loops were used resulting in 509 × 65 × 65 × 65 possible combinations. Many of these were ruled out by steric hindrance or poor superposition of the anchor G's preceding and following the loop. Nevertheless 4418 molecular models have been generated reflecting a variety of parallel/antiparallel dispositions, loop lengths, syn/anti glycosidic bond angles. Many of these models still suffered from long bonds resulting from merging fragments and steric hindrances and for this reason they were refined by energy minimization.

#### Molecular mechanics refinement

All 4418 model were subjected to 300 steps of molecular mechanics minimization keeping the G-tetrads fixed. At the end the energy distribution of the models was quite homogeneous with energies ranging mostly between 700 and 1500 kcal/mol. Only four models were clearly separated from the remaining ones at much higher energy (two at ca. 13000 kcal/mol and two at ca. 63000 kcal/mol) pointing out serious steric hindrance. Visual examination shows that the rebuilt G-tetrads are too close for these four models. The latter models have not been considered in the following clustering procedure.

#### Clustering of structural models

All energy minimized models are available, together with sequences and a table of energies and topologies, from the authors. However, for more convenient usage, the models were clustered according to unique glycosidic bond conformation, topology and loop length. The models sharing the same glycosidic bond conformations, topology and loop lengths were pairwise compared and for each model a threshold RMSD was chosen and a weight was assigned based on the RMSD with all other structures. The model with largest weight was chosen as representative of all models with RMSD lower than threshold. The procedure was repeated, increasing the threshold RMSD, until a single model was left. The energies of the resulting models range between 690 and 1656 kcal/mol, a range comparable with that found for experimental structures subjected to the same minimization procedure (590 to 913 kcal/mol), taking into account that experimental structures are typically already refined. The most representative structure for each of the 116 clusters (see Table 2) is provided [see Additional file 1].

**Table 2:** Features of modeled intrastrand G-quadruplexes.

**syn/anti**	**loop topology**	**strand polarity**	**loop 1**	**loop 2**	**loop 3**	**counts**
a a a	-p-p-p	ppp	1	1	1	10

a a a	-p-p-p	ppp	1	1	2	6

a a a	-p-p-p	ppp	1	1	3	53

a a a	-p-p-p	ppp	1	2	1	8

a a a	-p-p-p	ppp	1	2	2	2

a a a	-p-p-p	ppp	1	2	3	20

a a a	-p-p-l	ppa	1	2	3	6

a a a	-p-p-l	ppa	1	2	4	2

a a a	-p-p-p	ppp	1	3	1	58

a a a	-p-p-p	ppp	1	3	2	8

a a a	-p-p-p	ppp	1	3	3	237

a a a	-p-p-l	ppa	1	3	3	34

a a a	-p-l-l	pap	1	3	3	3

a a a	-p-p-l	ppa	1	3	4	12

a a a	-pd+p	paa	1	4	1	3

a a a	-pd+p	paa	1	4	2	10

a a a	-pd+l	ppa	1	4	3	10

a a a	-p-l-l	pap	1	4	3	1

a a a	-pd+p	paa	1	4	3	35

a a a	-p-p-p	ppp	2	1	1	4

a a a	-p-p-p	ppp	2	1	2	2

a a a	-p-p-p	ppp	2	1	3	21

a a a	-p-p-p	ppp	2	2	1	3

a a a	-p-p-p	ppp	2	2	2	1

a a a	-p-p-p	ppp	2	2	3	11

a a a	-p-p-l	ppa	2	2	3	3

a a a	-p-p-l	ppa	2	2	4	1

a a a	-p-p-p	ppp	2	3	1	23

a a a	-p-p-p	ppp	2	3	2	3

a a a	-p-p-l	ppa	2	3	3	17

a a a	-p-l-l	pap	2	3	3	2

a a a	-p-p-p	ppp	2	3	3	74

a a a	-p-p-l	ppa	2	3	4	5

a a a	-pd+p	paa	2	4	1	13

a a a	-pd+p	paa	2	4	2	3

a a a	-pd+p	paa	2	4	3	16

a a a	-pd+l	ppa	2	4	3	1

a a a	-p-l-l	pap	2	4	3	1

a a a	-p-p-p	ppp	3	1	1	28

s a s	-p-p-p	ppp	3	1	1	2

s s a	+l+p+p	aaa	3	1	1	34

s a a	-p-p-p	ppp	3	1	1	6

a a a	-p-p-p	ppp	3	1	2	10

s a a	-p-p-p	ppp	3	1	2	6

s s a	+l+p+p	aaa	3	1	2	7

s s a	+l+p+p	aaa	3	1	3	120

s a a	-p-p-l	ppa	3	1	3	12

a a a	-p-p-p	ppp	3	1	3	131

s s a	+l+p+l	paa	3	1	3	27

s a a	-p-p-p	ppp	3	1	3	49

a a a	-p-p-l	ppa	3	1	3	4

a a a	-p-p-l	ppa	3	1	4	1

s a a	-p-p-l	ppa	3	1	4	3

s s a	+l+p+l	paa	3	1	4	6

s s a	+l+p+p	aaa	3	2	1	10

s a a	-p-p-p	ppp	3	2	1	6

a a a	-p-p-p	ppp	3	2	1	9

s a a	-p-p-p	ppp	3	2	2	2

a a a	-p-p-p	ppp	3	2	2	3

s s a	+l+p+p	aaa	3	2	2	3

s a a	-p-p-p	ppp	3	2	3	22

a a a	-p-p-p	ppp	3	2	3	33

s s a	+l+p+p	aaa	3	2	3	46

s a a	-p-p-l	ppa	3	2	3	6

a a a	-p-p-l	ppa	3	2	3	9

s s a	+l+p+l	paa	3	2	3	9

s a a	-p-p-l	ppa	3	2	4	2

a a a	-p-p-l	ppa	3	2	4	3

s s a	+l+p+l	paa	3	2	4	3

s a a	-l-l-p	app	3	3	1	102

s s a	+l+p+p	aaa	3	3	1	102

a a a	-p-p-p	ppp	3	3	1	125

s a a	-p-p-p	ppp	3	3	1	80

a s a	-l-l-p	app	3	3	2	2

s a a	-l-l-p	app	3	3	2	70

s a a	-p-p-p	ppp	3	3	2	8

a a a	-p-p-p	ppp	3	3	2	9

s s a	+l+p+p	aaa	3	3	2	9

s a a	-l-l-l	apa	3	3	3	10

s s a	+l+l+l	apa	3	3	3	167

s a a	-p-p-p	ppp	3	3	3	241

a a a	-p-l-l	pap	3	3	3	30

s s a	+l+p+p	aaa	3	3	3	367

s a a	-l-l-p	app	3	3	3	385

a s a	-ld+l	aap	3	3	3	3

a a a	-p-p-p	ppp	3	3	3	450

s a a	-p-p-l	ppa	3	3	3	66

a a a	-p-p-l	ppa	3	3	3	68

a s a	-l-l-p	app	3	3	3	8

s a a	-p-l-l	pap	3	3	3	91

s s a	+l+p+l	paa	3	3	3	95

s a a	-p-p-l	ppa	3	3	4	12

s s a	+l+p+l	paa	3	3	4	15

a a a	-p-p-l	ppa	3	3	4	19

s a a	-l-l-l	apa	3	3	4	19

s s a	+ld-p	ppa	3	4	1	24

s a a	-pd+p	paa	3	4	1	6

a a a	-pd+p	paa	3	4	1	9

a a a	-pd+p	paa	3	4	2	15

s s a	+ld-p	ppa	3	4	2	3

s a a	-pd+p	paa	3	4	2	5

s a a	-pd+l	ppa	3	4	3	12

a a a	-pd+l	ppa	3	4	3	13

s a a	-p-l-l	pap	3	4	3	14

a a a	-p-l-l	pap	3	4	3	3

s s a	+ld-l	paa	3	4	3	3

s a a	-pd+p	paa	3	4	3	44

s s a	+ld-p	ppa	3	4	3	51

a a a	-pd+p	paa	3	4	3	80

s s a	+l+l+l	apa	3	4	3	82

s a a	-l-l-p	app	4	3	1	2

s a a	-l-l-p	app	4	3	2	8

a s a	-ld+l	aap	4	3	3	2

s a a	-l-l-p	app	4	3	3	33

a s a	d+pd	aap	4	3	4	114

s a a	d+pd	aap	4	3	4	8

The notation here follows Webba da Silva [56]. syn/anti indicates the conformation at the glycosidic bond of the first three G's. The loop topology is indicated by letters p (parallel), l (lateral) and d (diagonal) preceded by + or - sign to indicate clockwise or anti clockwise rotation when the first strand is progressing towards the viewer (see Figure 1). Similarly, the parallel or antiparallel (a/p) strand polarity in column 2 is with reference to the first strand and the order is according to the position in the quadruplex (rotating anti-clockwise with the first strand progressing towards the viewer), and in general not according to sequence order. The next three fields indicate loop lengths and the last field indicate the number of built models found with these features.

### Analysis of G-quadruplex models

#### Comparison with experimental structures

An obvious test for the methodology is to check whether it is able to recover the observed intrastrand G-quadruplexes from the fragments which are not taken from that intrastrand G-quadruplexes. Due to sequence diversity it will be in general hard to recover exactly the same sequence. For instance, for loops of length 2 there are only two fragments with different sequence. When one of the two loops is excluded from the list of fragments there will be no possibility to obtain a loop of length 2 with the same sequence. Nevertheless we will consider here the topologies which are generated from assembly of fragments with the same loop lengths.

In order to test the overall reliability of the method we considered the set of intrastrand structures with three G stem strands with loops of length 3. In the following we indicate the overall topology of a model by noting the sequence of loops as lateral (l), propeller (p) or diagonal (d). The clockwise (+) or anti-clockwise (-) rotation of lateral and propeller loops is with respect to a common frame of reference (see [56]). It was not possible to extend this analysis to the other structures because they contain loops of length 2 for which only two fragments are present in the dataset.

The structures with pdb id. 1KF1, 2GKU, 2HY9, 2JPZ, 2JSL, 2JSM and 3CDM all contain the core sequence GGGTTAGGGTTAGGGTTAGGG and adopt three different topologies: namely -p-p-p, -l-l-p, -p-l-l.

**Figure 2:**
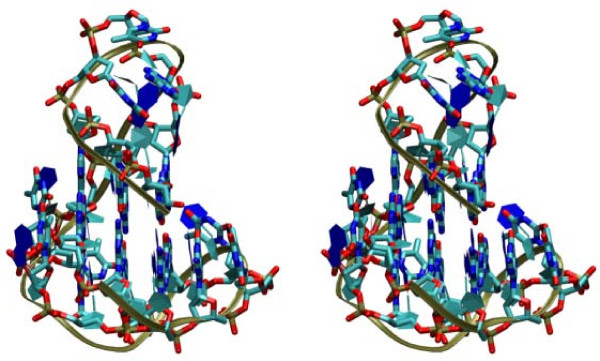
**Model for a human telomeric DNA G-quadruplex structure **(pdb id: 2HY9). In the stereoview the experimental structure is displayed as a ribbon with sugars and bases schematic representations and the model is displayed as solid bonds. The RMSD computed on all backbone atoms is 2.2 Å.

We considered for each topology all built models which do not contain any fragment derived from structures with the same topology. Moreover, due to the fragment clustering procedure adopted, no fragment is present in the dataset closely related to those present in those structures. All three topologies are actually represented several times in the built models with RMSD over sugars and phosphates from the original PDB structures between 2.0 and 3.0 Å. An example with the real structure (pdb id: 2hy9) and the model assembled from fragments is reported in Figure 2. Although not all loops are similar to the real ones, by construction, the topology and overall conformation is reproduced well by the model. The RMSD computed on all backbone atoms is 2.2 Å.

It must be however noted that not only the observed topologies for the sequence G_3_N_3_G_3_N_3_G_3_N_3_G_3 _are found in the models but also others, although the observed topology is among the most represented ones.

#### Topologies and loop lengths

Another test for the methodology is to check whether the relationship between loop lengths and topology matches the available experimental evidences.

Overall, the topologies of the models generated using all the available fragments are not evenly distributed (Table 3). Only 14 out of 26 possible looping topologies are found. There is a clear predominance of the -p-p-p topology that is found in ca. one third of all models.

**Table 3:** Topology distribution of model G-quadruplexes.

**topology**	**counts**
-p-p-p	1764

+l+p+p	698

-l-l-p	610

-p-p-l	285

+l+l+l	249

-pd+p	239

+l+p+l	155

-p-l-l	145

d+pd	122

+ld-p	78

-pd+l	36

-l-l-l	29

-ld+l	5

+ld-l	3

total number of topologies	total number of models

14	4418

The distribution of topologies (independent of glycosidic bond conformation and loop lengths) of all 4418 models is reported. The notation here follows Webba da Silva [56]. p, l and d stand for propeller-like, lateral or diagonal loop. Theand + signs refer to anti-clockwise or clockwise rotation of the loop around the G-quadruplex stem, respectively, when the first strand is progressing towards the viewer (see Figure 1).

This finding is consistent with the observation that the number of parallel fragments which are actually used for assemblying the stems of G-quadruplex is larger than the number of antiparallel fragments, although the starting fragments (i.e. strand-loop-strand fragments) do not show such parallel predominance. This might reflect a general more regular arrangement of parallel versus antiparallel strands, at least in the selected dataset. The adoption of a -p-p-p topology leads to right-handedness of the polynucleotide chain in the G-quadruplex.

Other well represented topologies are the mixed topologies +l+p+p, -l-l-p, -p-p-l, -pd+p, +l+p+l d+pd and the all-antiparallel +l+l+l.

Some care must be taken when considering potential G-quadruplexes involving loops of 4 residues because no propeller-like loop is found in the starting dataset of fragments. There are therefore no all parallel topologies involving loops of 4 residues, although it has been shown that the G-rich sequence in the human VEGF gene promoter adopts an all-parallel structure involving a loop of 4 residues [15,57]. Most frequently diagonal fragments are found.

Only 47 possible combinations of loop lengths are found out of 64 possible. Of these some are more largely represented as a consequence of the uneven distribution of the number of starting loops. In general there is no direct relationship between loop lengths and topology, although sequences with loops as short as 1 or 2 nucleotides are, as expected from the starting fragments, found in all-parallel topology. For longer loop lengths typically many different topologies are found.

It is interesting to note that some of the combination of loop lengths are found with unique topology, among these the most widely represented are 4, 3 and 4 (122 models), 1, 3 and 1 (58 models), 1, 1 and 3 (53 models) (Table 2).

The effect of loop length on G-quadruplex topology and/or stability has been studied by many authors [34,35,37-39,58-61] under different conditions. Not all studies however address the formation of intramolecular G-quadruplexes. The two recent papers by Bugaut and Balasubramanian [37] and Smargiasso et al. [38] investigate systematically the effect of loop length, with randomized sequences, on G-quadruplex stability and topology. In both studies intramolecular vs. intermolecular G-quadruplex formation is experimentally addressed. Notwithstanding different experimental conditions, these studies provide, among other results, a general conclusion which is well in line with previous evidences: in general short loops (and in particular the presence of loops of length one) strongly favor parallel arrangement of the strands while for longer loops antiparallel and mixed arrangements are observed. The topology of the models built here appears consistent with experimental evidences.

### Possible applications

The present study constitutes a proof of principle, obviously physical or statistical effective energy functions should more accurately measure the stability of the predictive models. Moreover the limited diversity in sequence does not allow to build models for all possible sequences. A third limitation of the present approach is that no consideration of flanking residues which are known to be important for the stability of G-quadruplex is taken into account. In addition to these problems the starting fragments are in limited number as exemplified by the lack of parallel propeller loops of length 4.

It is worth however to explore how structural predictions could complement experimental and bioinformatics approaches.

It must be clear that the actual structure adopted by a DNA sequence depends on many factors including flanking and loop sequences and environmental conditions. The models built from experimental fragments constitute however a set of structures whose features are consistent with experimental structures. Due to the limited number of structures solved so far, the set is not expected to cover all possible structures. However, even in the presence of polymorphism the models proposed here constitute structural working hypotheses that can complement experimental techniques.

The aim of the following subsection is to show, by selecting a few possible applications that inferences based on the built models are consistent with experimental evidence and thus provide an overall test of reliability for the proposed models.

It is well known that potential G-quadruplex sequences play a regulatory role but the nature of such role is different according to the position of the sequence with respect to the TSS and the strand where it is found [43].

The models provided by the present study could be used straightforwardly as starting models for molecular dynamics simulations or docking studies. Another possibility is to use the topology information provided here to complement other studies. The same topology could be required by different DNA quadruplex sharing a common mechanism of gene regulation. We consider here that among loop length combination showing a unique (all-parallel -p-p-p) conformation we find loop length combination of (1,1,1), (1,2,1) and (1,3,1). The second one has been described experimentally as an all-parallel G-quadruplex [62], while the other two have not been solved experimentally. For the first one the all parallel topology should be strongly favored by the presence of all single-nucleotide loops [36-38]. For the last combination of loop lengths also a mixed topology is in principle possible, but it is not found among our models, notwithstanding the large number of loops of length 3 available among the starting fragments.

A (1,3,1) loop length combination has been found in the promoter of the oncogene RET and its topology was described as all-parallel consistent with our predictive model [63].

A word of caution is due here: although intramolecular G-quadruplex formation has been observed for this loop length combinationation [37], a study by Vorlickova [35] and colleagues tested under different conditions sequences (G_3_X_*n*_)_3_G_3 _with *n *= 1, 2, 3 and they found that these sequences formed mostly intermolecular G-quadruplexes. Only in ethanol solutions the same sequences adopted intramolecular parallel conformation. In this work we do not consider intermolecular, but only intramolecular G-quadruplexes. Moreover, in view of the known polymorphism of G-quadruplexes, our models suggest which conformation could be attained by a given sequence, compatible with structural observations. These conformations could be adopted only under peculiar environmental conditions, e.g. such as those described by Vorlickova and coworkers [35] or Bugaut and Balasubramanian [37].

In the following we will focus on the G-quadruplex forming sequence found in the RET promoter, whose loop length combination is associated with a unique all-parallel (-p-p-p) topology, and with all loop length combinations sharing the latter feature in our models.

#### Molecular dynamics simulations

An obvious application of structural models is computer simulations of their molecular dynamics. A necessary, albeit not sufficient, condition for a model to be accurate is that the structure is stable during a molecular dynamics simulation for a time sufficient in principle to develop major conformational rearrangements. The benefits and limits of molecular dynamics simulations of G-quadruplexes have been reviewed by Spooner and Spackova [64]. In the study by Hazel et al. [58] molecular dynamics simulations complemented experiments and model building was performed in order to provide starting models. We consider here as an example the sequence GGGCGGGGCGGGGCGGG that is found in the promoter of the oncogene RET, which adopts an all-parallel topology [63].

The most representative predictive model for the unique all-parallel (-p-p-p) topology for loop length combination (1,3,1) was taken and the sequence was mutated to the target sequence. Two potassium ions were added at the centre of the O6 atoms of adjacent tetrads and counterions were further added to make the system neutral. The system was solvated in a box of water extending at least 12 Å away from each heavy atom of the solute. The preparation of the system is essentially as previously described for a different system [65]. The forcefield employed is CHARMM version 31 [66,67].

Molecular dynamics simulation was run for 20 ns in order to check for any major conformational change which could indicate bad quality of the starting model or wrong topology [58,64].

After few hundred picoseconds one of the two potassium ions at the centre of adjacent tetrads goes in solution while the other is firmly retained. Loss of ions from the central channel has been observed before in molecular dynamics simulations and it has been ascribed to forcefield inaccuracies [64]. During the simulation the G-quadruplex structure is mantained. The average RMSD from the starting structure is 1.0 Å. Larger fluctuations are observed at the three residue loop both for the backbone and for the base moieties similar to other molecular dynamics simulation studies [58,64]. No loop residue is involved in intramolecular hydrogen bonds. This example proves (at least on the timescale of 20 ns) that the model quality is suitable for molecular dynamics simulations because otherwise large changes in the G-quadruplex structure would be expected [58,64].

#### Docking studies

Predictive models of G4 may be employed for docking studies (see e.g. [68,69]). As an example we considered the model for the sequence GGGCGGGGCGGGGCGGG that is found in the promoter of the oncogene RET, which adopts an all-parallel topology [63], as in the previous section.

This sequence has been shown to be stabilized by the cationic porphyrin TMPyP4 (5,10,15,20-tetrakis(1-methylpyridin-1-ium-4-yl)-21,22-dihydroporphyrin) and it was suggested that the binding involves stacking rather than intercalation [63].

Two G-quadruplex-TmPyP4 complexes have been structurally characterised by NMR (pdb id. 2A5R, [70]) and by X-ray crystallography (pdb id. 2HRI, [71]). The two complexes show remarkable differences. In the NMR structure the porphyrin is stacking over the first tetrad and is covered by the two residues 5' to the G-quadruplex. In the crystal structure one porphyrin is stacked over a base pair over the tetrads and the other is contacting a grove with electrostatic interaction with a phosphate and a stacking interaction with a base in the loop.

**Figure 3:**
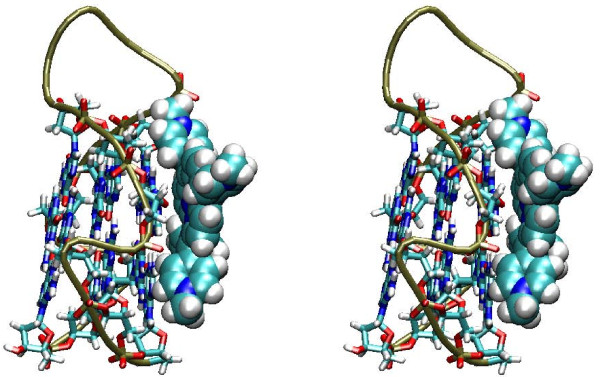
**Model of TMPyP4 docking on the model for RET promoter G-quadruplex structure**. In the stereoview TMPyP4 atoms are shown as Van der Waals spheres and DNA backbone is shown as a tube. The bonds of residues of the G-quadruplex tetrads are shown.

The ligand structure was taken from the Hic-Up server [72]. Based on the SMILES representation of the compound available from the database PubChem (CID: 4234) [73] partial charges have been assigned by the program Babel [74] implementing the Gasteiger and Marsili method [75]. The structure of the model G-quadruplex with partial charges has been obtained as decribed in the previous section. The program Dock6.3 [76] has been used for generating, scoring and clustering TMPyP4-G-quadruplex complexes following a standard protocol and using the AMBER forcefield for estimating the energy of van der Waals intermolecular contacts. 20000 poses were generated and after clustering at 2.0 Å RMSD the best 10 were retained, showing all large negative interaction energies. Consistent with the above cited previous studies, in nine out of ten complexes the arrangement of the prophyrin is parallel and stacking onto the tetrads, although stacking involves only half of the tetrad (Figure 3). In the remaining complex the porphyrin is contacting the G-quadruplex in the groove and displays electrostatic interaction between the pyrimidinium and the phosphate.

Overall these results are consistent with what could be expected based on previous structural characterization and thus show that the models can provide a starting target for use in docking studies.

#### Cancer genes

Potential G-quadruplex forming sequences have been found in a number of cancer genes [33] mostly sharing the first and last loops of length 1.

Consistent with earlier studies [33,37,38], sequences having the second loop of length 1 to 3 can adopt in our models only an all-parallel -p-p-p topology, while any other topology would be not consistent with the available set of experimental structures. We further explore whether the presence of sequences for which a unique all-parallel topology is found could be a distinctive feature of cancer genes. Following previous analyses we have limited our search to a putative regulatory region of -200 bp, 0 bp around the TSS of all genes in the Ensembl database. We first aligned the sequences:

G_3_N_1_G_3_N_1_G_3_N_1_G_3_,

G_3_N_1_G_3_N_2_G_3_N_1_G_3_,

G_3_N_1_G_3_N_3_G_3_N_1_G_3_

on the putative regulatory regions and the search returned 728 unique genes containing at least one of the sequences.

Before any further consideration it should be considered that the pattern G_3_N_1_G_3 _is shared with consensus motif G_3_CG_3 _of SP1 binding site, which is rather common in human genes in the region within 200 bp upstream of the TSS [77]. Inclusion of the G_3_CG_3 _consensus sequence for SP1 binding site in many of the potential G-quadruplex sequences is likely to add noise to any statistical analysis and to reduce the calculated significance values.

The Ensembl gene names were translated, where possible, onto HNGC gene names and the overlap between the set of the resulting 686 genes and the Census set of 385 cancer genes available at the [78,79] was determined. The overlap set contained 23 genes, a number higher than that expected by chance, i.e. 14.5.

The probability that 686 genes chosen randomly out of 19589 genes with HGNC name could have an overlap of 23 or more with the Census set (385 genes) was calculated using the hypergeometric distribution and the resulting p-value was 0.01. This result, based on putative adoption of a common structure, suggests that G-quadruplex gene regulation may be a common feature of cancer genes.

The above set of loop length combinations is however only a restricted set of the larger set of all loop length combinations which are associated in the predicted models to a unique all-parallel (-p-p-p) topology (see Table 2).

The same analysis has been repeated considering the latter set. If the topology is an important feature shared by G-quadruplex sequences found in many oncogenes we would expect also for the larger set of loop combinations a higher number of hits in oncongenes than expected by chance. Indeed this is the case. The overlap between the 1607 genes, containing at least one of the selected loop combinations, and the Census set consists of 47 genes (Table 4), higher than the expected 32, and corresponding to a p-value of 0.003. These results are consistent with the known importance of all-parallel topology for G-quadruplex forming sequences in the regulation of proto-oncogenes [33].

**Table 4:** Human cancer genes containing potential all-parallel G-quadruplexes.

**Gene symbol**	**Gene name**
AKT1	v-akt murine thymoma viral oncogene homolog 1

ASPSCR1	alveolar soft part sarcoma chromosome region, candidate 1

ATF1	activating transcription factor 1

BCL3	B-cell CLL/lymphoma 3

BRCA2	familial breast/ovarian cancer gene 2

CARD11	caspase recruitment domain family, member 11

CDH11	cadherin 11, type 2, OB-cadherin (osteoblast)

CLTCL1	clathrin, heavy polypeptide-like 1

ELN	elastin

EPS15	epidermal growth factor receptor pathway substrate 15 (AF1p)

ERCC2	excision repair cross-complementing rodent repair deficiency complementation group 2 (xeroderma pigmentosum D)

ETV6	ets variant gene 6 (TEL oncogene)

FGFR3	fibroblast growth factor receptor 3

FNBP1	formin binding protein 1 (FBP17)

FOXP1	forkhead box P1

FSTL3	follistatin-like 3 (secreted glycoprotein)

GATA1	GATA binding protein 1 (globin transcription factor 1)

HIP1	huntingtin interacting protein 1

HOXA11	homeo box A11

HOXA13	homeo box A13

HOXA9	homeo box A9

IGK@	immunoglobulin kappa locus

IRF4	interferon regulatory factor 4

JAZF1	juxtaposed with another zinc finger gene 1

LHFP	lipoma HMGIC fusion partner

MLLT6	myeloid/lymphoid or mixed-lineage leukemia (trithorax homolog, Drosophila); translocated to, 6 (AF17)

MSI2	musashi homolog 2 (Drosophila)

MSN	moesin

MUC1	mucin 1, transmembrane

MYCL1	v-myc myelocytomatosis viral oncogene homolog 1, lung carcinoma derived (avian)

MYCN	v-myc myelocytomatosis viral related oncogene, neuroblastoma derived (avian)

MYC	v-myc myelocytomatosis viral oncogene homolog (avian)

PIM1	pim-1 oncogene

POU2AF1	POU domain, class 2, associating factor 1 (OBF1)

PTEN	phosphatase and tensin homolog gene

RANBP17	RAN binding protein 17

RAP1GDS1	RAP1, GTP-GDP dissociation stimulator 1

RET	ret proto-oncogene

SEPT6	septin 6

SFRS3	splicing factor, arginine/serine-rich 3

SS18L1	synovial sarcoma translocation gene on chromosome 18-like 1

TAF15	TAF15 RNA polymerase II, TATA box binding protein (TBP)-associated factor, 68 kDa

TCF12	transcription factor 12 (HTF4, helix-loop-helix transcription factors 4)

TMPRSS2	transmembrane protease, serine 2

TRIM33	tripartite motif-containing 33 (PTC7, TIF1G)

TSHR	thyroid stimulating hormone receptor

ZNFN1A1	zinc finger protein, subfamily 1A, 1 (Ikaros)

#### Developmental genes

The restricted set of genes that contain a potential all-parallel quadruplex helix has been screened for overrepresentation in Gene Ontology annotation. The general terms "developmental process", "system development", "anatomical structure development", "multicellular organismal development" are found with the lowest p-values (less than 10^-10^). The same analysis on the larger set of genes containing a potential all-parallel quadruplex helix gives essentially the same results with even lower p-values (ranging from 10^-12 ^to 10^-17^).

The same search for the all parallel motifs discussed above on the restricted set was performed on putative regulatory regions of -200 bp, 0 around the TSS of all mouse genes in the Ensembl database returning 841 genes. Remarkably enough, looking for overrepresented Gene Ontology terms in this set of genes we found exactly the same terms already found in the human case, albeit with slightly higher p-values (less than 10^-8^). Comparing the 728 human genes and the 841 mouse genes using the list of 21605 orthologous genes between human and mouse contained in the Ensembl database we found an intersection of 104 genes. The p-value of the overlap, computed under the assumption of no significant conservation in putative gene regulatory regions is as low as 10^-31^. However, since some conservation in the putative regulatory regions is expected and is indeed found the latter p-value should be regarded with some caution.

### Conclusion

A large number of molecular models has been generated for intrastrand G-quadruplex formed by G_3_X_*n*1_G_3_X_*n*2_G_3_X_*n*3_G_3 _sequences. For a given sequence not all topologies are possible with the available repertoire of fragments due to steric hindrance and low superimposability. Since all molecular models are generated by fragments coming from observed quadruplex structures, molecular models are in principle reliable and may be used for interpretation of experimental data. Molecular models for different loop length combinations suggest that the all-parallel topology is strongly favored. Notwithstanding the limitations of the approach, the models could be useful for molecular modeling and docking studies, and in general to complement other laboratory and bioinformatics methods.

## Methods

### DNA fragment generation

#### Quadruplex structure selection

Structures for DNA (or DNA/RNA or RNA) quadruplexes were selected using the search tools available at the nucleic acid databank [49] requiring "quadruple helix" as structural feature.

The search resulted in 101 entries. Of these 91 are containing only DNA quadruplexes. The PDB code for the 101 entries was used for retrieving the relevant structures from the Protein Data Bank [50].

This search was apparently missing some of the G-quadruplexes in the PDB. For this reasons we selected all structures in the PDB containg anywhere the words quadruplex or tetraplex and we hand filtered those that could contain a genuine DNA G-quadruplex. The latter step retrieved additional 51 structures. The selected structures were searched for the presence of strands with three adjacent G's paired with another strand with three adjacent G's with either parallel or antiparallel linear arrangement, and with loop connection of one to four nucleotides. The loop lenghts considered here are somewhat shorter than the limit of seven used e.g. by Chowdhury and coworkers [47]

Since the quadruplex are assembled from these fragments we found useful to reference these fragments and their pairs of bases defining the G-tetrad plane to a standard G-tetrad. The parallel or antiparallel orientation of the two G-strand is an obviously important feature of the fragment. Referencing serves the purpose of detecting and storing fragments that can be used for building the G-quadruplex and is not meant as a definition for classification. A standard for classification and notation of G-quadruplexes has been proposed by Webba da Silva [56]. We conform here to that proposed standard, although we report also a local description of the structure (vide infra)

A standard G-tetrad was generated by rotation and translation of a G base (taken from the standard fragments of the X3DNA program [80]). The best hydrogen bonding geometry was obtained by rotating repeatedly the G base of 90 degrees and traslating by -.70 and 7.10 Å along the x and y axis respectively, with reference to the coordinates used in the X3DNA base coordinates.

The first G base of each fragment with sequence G_3_X_*n*1_G_3 _was superimposed to base 1 in the model G-tetrad. Then the first (last) G base of the second run of three G's in the fragment was superimposed in turn onto the other bases in the G-tetrad in order to find the first base pair of the parallel (antiparallel) three G's pair. The same procedure with due modifications was repeated for the second and third G pairs. The fragment was accepted as good if the RMSD in all three superpositions was less than 1.0 Å. The tolerant threshold was dictated by the large conformational heterogeneity observed in G-tetrads.

The first (last) G base could be in anyone of the three other positions of the G-tetrad (Figure 1). The second and third G's could be found over or below the plane defined by the G-tetrad used for superposition of the first G depending on the torsion angle at the glycosidic bond.

For each fragment the loop and the "stem" constituted by two strands of 3 adjacent G's were stored. The 147 fragments (with the 147 loops and 147 stems) obtained in this way are redundant because the same structure may have been resolved by different groups and techniques and because a single PDB entry may contain the same structure more than once.

In order to remove redundancy we performed clustering. All fragments identical in sequence were compared and representatives were selected in such a way that none chosen conformation has less than 0.8 Å RMSD on all heavy atoms. This led to 68 unique complete fragments, 67 loops and 57 stems.

#### DNA G-quadruplex assembly from fragments

The 57 stems were used to assemble the quadruple helix. Three "stems" were assembled together by superimposing the second three G's with the first three G's of the next stem with less than 0.8 Å RMSD and with no overlap of heavy atoms at more than 0.5 times the sum of their van der Waals radii. This procedure led to 646 quadruplex stem models. The stems were therefore further modified by substituting the model G-tetrad for each G-tetrad. The model G-tetrad was set in place by superimposing first the G of the first strand and then superimposing the four C1' atoms to the closest ones in the G-tetrad. If the RMSD was larger than 3.0 Å the model was not taken into account. At the end of this step 509 models were retained. The conformation of the glycosidic bond angle is thus determined by the first strand of the quadruplex.

Finally loops were added to the quadruplexes whenever the superposition of the sugars linked to the G's preceding and following the loop gave an RMSD less than 0.8 Å and with no overlap of heavy atoms at more than 0.5 times the sum of their van der Waals radii. Although the combinatorial number of possible models is extremely large, in practice this computation may be performed on a PC. This last step generated 4418 models.

#### Molecular mechanics refinement

Due to the rather tolerant cutoff on RMSD's the bonds connecting stems and loops were in many instances large. The refinement was performed by first substituting the G-tetrads with the regular G-tetrad generated by optimal rototraslation of G, as described above, and then keeping the G-tetrads fixed and performing energy minimization. For this purpose the program NAMD [81] was used employing a dielectric constant of 10 and the forcefield CHARMM version 31 [66,67]. 300 steps of conjugate gradients minimization were performed keeping the base atoms of the tetrads fixed.

#### Clustering of structural models

All energy minimized models sharing the same glycosidic bond conformations, topology and loop lengths were clustered in separate groups. All models within a single group were pairwise compared. A threshold RMSD *t *was chosen and a weight *w*_*i *_was assigned to each model *i *based on the RMSDs lower than *t *with all other structures:



The model with the largest weight was chosen as representative of all models with RMSD lower than threshold. The procedure was repeated doubling progressively the threshold starting from 0.4 Å until a single model was left.

#### Genomic searches and analysis

All regions 200 bp upstream the ranscription start site (TSS) of all human genes for all transcripts have been downloaded from Biomart site . The database and the dataset were ENSEMBL 53 GENES and NCBI36i respectively. The search for potential G-quadruplex sequences with proper loop lengths was performed using the program glsearch in the fasta35.1 software package . The same analysis was repeated for all mouse genes using the same database and the dataset NCBI37.

The list of orthologous genes was obtained from the Biomart site selecting only protein-coding genes.

The Census set of 385 cancer genes was downloaded from the [78,79].

In order to evaluate the significance of the overlap of k genes between two given sets of n and m genes both taken from the same set of N genes we estimated the probability (p-value) that an equal or larger overlap set could be obtained by chance.

This probability is computed using the hypergeometric distribution:



## Authors' contributions

FF and HH carried out the implementation and computational analysis. AC, GE, PV carried out structural analysis. MC and DC wrote most programs for genomic analysis and participated designing genomic analysis. LEX participated in designing the study and preparing the manuscript. All authors read and approved the final manuscript.

## Supplementary Material

Additional file 1 **Models for intrastrand G-quadruplexes**. The name of the file contains all topology information. Each field is separated by the underscore character. The notation here follows Webba da Silva [56]. The first field indicates the glycosidic bond conformation in the first G-quadruplex strand a stands for *anti *and s stands for *syn*. The second field indicates the loop topology by letters p (parallel), l (lateral) and d (diagonal) preceded by + or - sign to indicate clockwise or anti clockwise rotation when the first strand is progressing towards the viewer. Similarly, the third field indicates the parallel or antiparallel (a/p) strand polarity with reference to the first strand. The order is according to the position in the quadruplex (rotating anti-clockwise with the first strand progressing towards the viewer), and in general not according to sequence order. The next three fields indicate loop lengths. The .nrg files contain the energy as ouput by the program NAMD [81]. The total energy is reported in the twelfth field. The .fas files contain the sequence of the representative model. Click here for file
